# Why do poxviruses still matter?

**DOI:** 10.1186/s13578-021-00610-8

**Published:** 2021-05-22

**Authors:** Zhilong Yang, Mark Gray, Lake Winter

**Affiliations:** 1 Department of Veterinary Pathobiology, College of Veterinary Medicine & Biomedical Sciences, Texas A&M University, College Station, TX USA; 2 Division of Biology, Kansas State University, Manhattan, KS USA

**Keywords:** Poxvirus, Vaccinia virus, Virology, Smallpox, Vaccine vector, Oncolytic therapy, Biodefense, Public health, Animal health

## Abstract

Poxviruses comprise many members that infect both vertebrate and invertebrate animals, including humans. Despite the eradication of the historically notorious smallpox, poxviruses remain significant public health concerns and serious endemic diseases. This short review briefly summarizes the present, historical, and future threats posed by poxviruses to public health, wildlife and domestic animals, the role poxviruses have played in shaping modern medicine and biomedical sciences, the insight poxviruses have provided into complex life processes, and the utility of poxviruses in biotechniques and in fighting other infectious diseases and cancers. It is anticipated that readers will appreciate the great merit and need for continued strong support of poxvirus research; research which benefits not only the expansion of fundamental biological knowledge but also the battle against diverse diseases.

## Background

The poxvirus family is a large family of double-stranded DNA viruses designated *Poxviridae*. Based on the International Committee on Taxonomy of Viruses (ICTV) Taxonomy 2019 release [1], The *Poxviridae* comprises two subfamilies: *Chordopoxvirinae* (18 genera, 52 species) and *Entomopoxvarinae* (4 genera, 30 species). Poxviruses infect a range of animals including insects, birds, reptiles, marsupials, and mammals [2, 3], and cause disease in many, including humans. In this review, we will briefly discuss the historical significance and research endeavors pertaining to poxviruses. We will then focus on why it is critical that we expand our understanding of these viruses.

## Poxviruses have historical, current, and future significance to public health

**Fig. 1:**
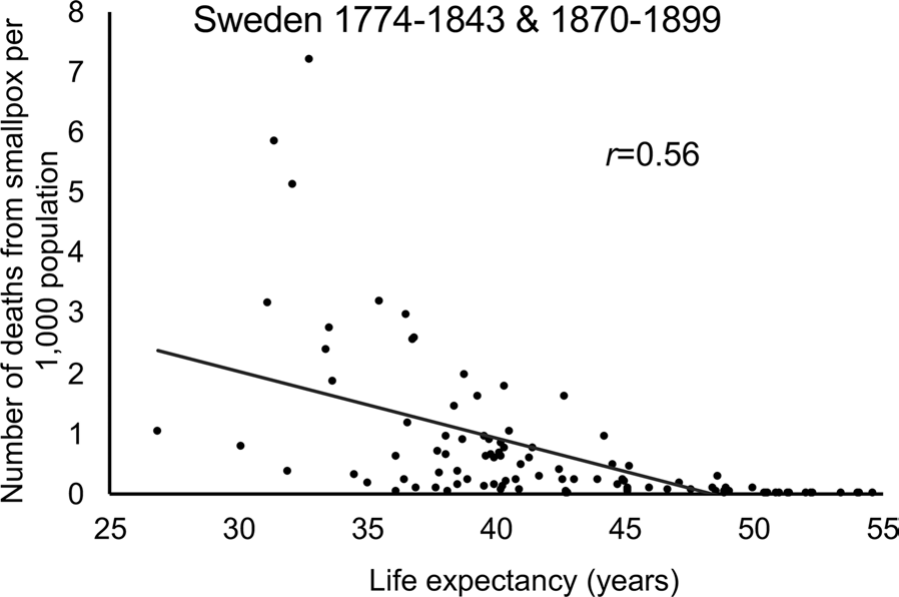
Correlation coefficiency of life expectancy and number of deaths from smallpox per 1000 population from 1774 to 1899 in Sweden. Shown data points are for years available. Data was extracted from https://ourworldindata.org/smallpox

Smallpox, caused by variola virus of the orthopoxvirus genera, is one of the most, if not the most, deadly and dreaded diseases in human history. While the origin of smallpox is unknown, it is believed that variola virus evolved from a rodent poxvirus [4]. While the lesions found on the mummy of Egyptian Pharaoh Ramesses V (died 1145 BC) suggest that smallpox existed over 3000 years ago [5, 6], the earliest written documentation of smallpox-like symptoms were from Ge Hong’s (283–343, Eastern Jin Dynasty, China) book, *Zhouhou Beiji* *Fan*, or, *Handbook of Prescriptions for Emergency*. Smallpox has likely caused more human deaths than all other infectious diseases combined. According to an estimation by Crosby et al., 10–15 million people had been infected with smallpox in the year of 1967 [7]. With a mortality rate of 30–40%, smallpox killed approximately 300 million people in the twentieth century alone [8]. Considering smallpox was eradicated in 1980, this estimation suggests approximately 5 million smallpox deaths per year during the endemic phase in the early years of the twentieth century. Notably, a negative correlation between human life expectancy and smallpox outbreaks was observed in Sweden between year 1774 and 1843 (Fig. 1, data from https://ourworldindata.org/smallpox) [9], when the date are available. This historic mortality has resulted in Smallpox outbreaks reshaping the history of many countries. For example*,* Emperor Kangxi (1654–1722), who ruled for over 60 years during the Qing Dynasty, substantially influenced Chinese history. After he suffered from smallpox and survived, his immunity is believed to have aided in him becoming emperor [10].

While the World Health Organization (WHO) declared smallpox eradicated in 1980 following a world-wide vaccination campaign [11], concerns persist that smallpox may re-emerge accidently from forgotten stocks of the virus, from de novo synthesis, or from melting permafrost due to global warming [12, 13]. This is especially worrisome because of the low worldwide vaccination rate against smallpox since 1980, reducing rates of smallpox immunity in the population. The US military considers the risk of weaponized smallpox sufficient to justify the continued vaccination of all its military personal against the disease [14]. While smallpox had caused countless human lives historically and they continue to pose threats to public health, only recently did the U.S. FDA approved TPOXX as the first specific smallpox drug [15]. TPOXX targets a major viral protein that is important for morphogenesis (i.e. F13 in vaccinia virus) [16]. Cidofovir has off-label use for poxvirus infection and was originally approved for AIDS-related retinal cytomegalovirus infection [17–19]. Drug-resistance and side effects are still the concerns [20], promoting the necessity to search for new drugs. In fact, the U.S. Biomedical Advanced Research and Development Authority of the federal HHS Office of the Assistant Secretary for Preparedness and Response (BARDA) is actively seeking a second smallpox drug for clinical development, according to a recommendation from the Institute of Medicine od the U.S. [21, 22].

Humans are the exclusive host of variola virus. Another poxvirus, molluscum contagiosum virus, also exclusively infects humans. Molluscum contagiosum accounts for 1 in 500 outpatient visits in the US and is particularly common in young people [23]. The virus causes self-limiting benign tumors in healthy people but can cause serious complications in immune-compromised individuals [23]. Besides these two human-specific poxviruses, many animal poxviruses can cause severe diseases in humans, and some others may potentially be the pathogens for diseases with unidentified causes. An ectromelia virus, erythromelalgia-associated poxvirus, or ERPV, was isolated from patients among school-age children in rural China. The disease is characterized by pharyngitis followed by burning pain and inflammation in the extremities, with seasonal outbreaks occurring every few years in spring or winter [24–26]. While whether ERPV is the cause of the epidemic erythromelalgia is still unclear and needs further investigation, some other poxviruses clearly cause human infections. For example, ORF virus causes sore mouth disease in goats and sheep, but can also infect humans. Cowpox virus can elicit serious illnesses in human hosts. A novel orthopoxvirus recently caused infections in humans in the state of Alaska and is believed to have been transmitted by animals [27, 28]. Monkeypox is endemic in Africa and has a 10% death rate among the humans it infects [2]. A study published in 2010 showed that monkeypox virus cases have increased over 20-fold in the Democratic Republic of Congo (DRC) during the 30 years following the termination of the smallpox vaccination campaign, as smallpox vaccine can protect humans from monkeypox infection. Individuals vaccinated against smallpox were at a 5.2-fold lower risk of contracting monkeypox compared to unvaccinated individuals [29]. New geographic areas reporting monkeypox cases have increased during recent years and now include the Central African Republic, DRC, Liberia, Nigeria, and Sierra Leone. In DRC, more than 1,000 suspected cases have been reported each year since 2005 [30]. According to the WHO, a total of 4,594 suspected cases of monkeypox were reported in the DRC from 1 January through 13 September 2020 (https://www.who.int/csr/don/01-october-2020-monkeypox-drc/en/). The numbers are likely underestimated due to a lack of reliable surveillance systems. Further spread of monkeypox is a real concern. In the last few years, several outbreaks of monkeypox have occurred in Central and Western Africa, and travel-related monkeypox cases have been reported in the United Kingdom, Israel, and Singapore [30–34]. An outbreak occurred in the USA in 2003 that was eventually traced to animals imported from Africa [35].

Among the numerous poxviruses that can infect animals, many have significant impacts on economically important livestock and ecologically endangered wildlife. Capripoxviruses cause lumpy skin disease (LSD) in cattle, sheep (sheeppox), and goats (goatpox), and can cause significant economic losses in commercial herds of these animals. Recent outbreaks of capripoxviruses in Asia indicate the expansion of the viruses into new regions in addition to Africa and the Middle East [36]. With increasing global trade, it would not be surprising to see these viruses continue to expand their geographic range. In addition to mammals, avipoxviruses impact hundreds of domestic and wild bird species, including chickens, turkeys, penguins, and songbirds, risking economic loss and endangering wildlife [37, 38]. Other examples of poxvirus diseases include swinepox, salmon gillpox, and crocodile pox [2].

## Poxviruses have shaped modern medicine and biomedical science

The battle against smallpox made important contributions to the development of modern medicine and the biomedical sciences. Edward Jenner, the famous English physician and scientist, carried out pioneering work to combat infectious disease through vaccination, applying a scientific approach to preventing smallpox prior to the widespread acceptance of germ theory. In 1980, one hundred and eight-two years after Jenner published his work *An inquiry into the causes and effects of the variolae vaccinae* in 1798, the WHO declared the eradication of smallpox through a global vaccination campaign [11]. As of 2021, smallpox is the first and only human disease to have been eradicated by deliberate vaccination. The great success of this evidence-based scientific approach to eradicate a specific disease greatly increased people’s acceptance of modern biomedical practices and many believe that smallpox eradication remains the most important achievement of modern medicine.

Jenner’s work on smallpox vaccination had profound influence on the development of many core branches of today’s biomedical sciences. The impact of his work on modern vaccinology is apparent, as there are numerous human vaccines available today, protecting us from over two dozen infectious diseases. There are also many veterinary vaccines now in use. It is particularly remarkable that multiple severe acute respiratory syndrome coronavirus 2 (SARS-CoV-2) vaccines have been successfully developed and approved for use, all within one year of the discovery of the pathogen responsible for the COVID-19 pandemic. Although Jenner’s work preceded the germ theory of infectious disease and immunity theory after infection, his successful smallpox vaccination work provided the foundation for these groundbreaking theories to gain acceptance. As a result, Jenner is now widely regarded as the founding father of immunology [39].

As the vaccine used to eradicate smallpox and the prototypic member of poxviruses, vaccinia virus has become the most extensively studied virus in the *Poxviridae* family, contributing many critical concepts to the development of modern virology and molecular biology. For example, the publication, *Studies on the Cultivation of the Virus of Vaccinia*, by Steinhardt and Lambert in 1913 documented the first animal virus successfully grown in tissue culture [40]. The production of viruses in tissue culture has proven fundamental to the research underpinning virtually all modern virology. Vaccinia virus went on to be used to elucidate the virion containing transcription machinery and the 5’ cap structure of eukaryotic mRNAs to name only two examples of the paradigm shifts in the field of virology (and more broadly in biology) that would not have been possible before [41–44].

## Poxviruses provide ample opportunities to understand complex life processes

Of the dozens of poxviruses, each has a DNA genome encoding hundreds of genes that support a complex replication cycle [3]. Many of the poxviruses’ encoded enzymes are utilized as tools for molecular biology and biotechniques. A number of commercialized biotechnology products have been successfully developed based on enzymes identified from vaccinia virus, exemplified by the vaccinia capping system for in vitro synthesized RNA and TOPO cloning which is based on vaccinia DNA topoisomerase [45, 46]. It will not be surprising to see more poxvirus-based biotechnology products in the future. In addition, although poxviruses are “old” viruses, many mechanisms of their replication cycle are still poorly understood, partially because of their complex virion and life cycle. For example, vaccinia virus needs a complex comprising 11 proteins for cell fusion and entry [47]; the most complex fusion-entry system among known mammalian viruses. The mechanism of entry is still largely unknown. Many other aspects of poxvirus virion assembly, membrane morphogenesis, and egress are also still poorly understood. Therefore, poxviruses provide phenomenal scientific opportunities for investigators in the field of virology.

Although poxviruses encode hundreds of genes, similar to other viruses, they still rely on host cells to complete their replication cycle and have complex interactions with their hosts. Eukaryotic cells, including mammalian cells, have a well-developed innate immune system to detect, respond to, and limit viral pathogens. It is estimated that more than one third of the over 200 vaccinia virus-encoded genes are dedicated to modulating innate immune response as a strategy to evade host antiviral immunity. Vaccinia’s viral proteins can counteract almost all known innate immune mechanisms. An outstanding review by Smith et. al. summarized these vaccinia virus immunomodulatory proteins nicely [48]. More vaccinia virus innate immunomodulatory proteins continue to be described and characterized. For example, B2 and F17 use distinct mechanisms to evade the cGAS-STING DNA sensing pathway [49, 50]. Yet, the functions and mechanisms of action of many of these poxvirus immunomodulators are unknown. Many of the remaining poxvirus non-immunomodulators also interact with cellular processes to facilitate virus entry, DNA replication, gene expression, viral envelop membrane morphogenesis, assembly, egress, and spreading. Research on these poxvirus gene functions will provide molecular tools to decipher aspects of cellular processes, in addition to understanding viral replication strategies. Work in the author’s laboratory has recently focused on vaccinia virus factors that interact with host cell protein synthesis and metabolism machinery [51–57], with the rational that the study of virus interactions with these host house-keeping functions is critical to elucidate the poxvirus replication strategy and the associated fundamental cellular processes.

## Employing poxviruses to fight other diseases

Poxviruses are broadly used as tools to fight many other diseases. They are being developed as vaccine vectors for other infectious diseases, oncolytic therapeutics for many cancers, gene delivery vehicles, and protein expression systems in mammalian cells. Several inherent biological features of poxviruses render them extremely effective as vaccine and gene-delivery vectors and as oncolytic agents. First, poxviruses have large genomes, which allow large foreign DNA fragment (over 25 kb), containing multiple genes, to be inserted in the viral genome for genetic engineering [58–60]. Second, the entire poxvirus replication cycle is in the cytoplasm and does not enter the nucleus [3], which provides poxviruses an advantage as vaccine vectors and oncolytic agents since the viral DNA is unlikely to be integrated into the host genome. Third, the poxvirus genes are expressed as a cascade with early, intermediate, and late classes and a broad range of expression levels [3, 61–63], which provides ample opportunity to modulate the expression timing and level of foreign genes. Fourth, plenty of data has been accumulated during the world-wide smallpox vaccination campaign, which provides an excellent safety baseline and is useful for further improving the safety profile. Fifth, poxvirus-vectored vaccines have the capability to induce both humoral and cellular immune responses to foreign antigens they carry [64]. Sixth, there are many poxviruses, including replicating and non-replicating ones in humans and other animals, allowing researchers to choose the most applicable. Seventh, poxviruses are stable, grow very fast, and can be produced at high titers for large scale manufacture. Lastly, when used in oncolytic therapy, poxvirus infection can stimulate anti-tumor immunity [65], which is critical to removing cancer cells.

Poxviruses began to be employed as vaccine vectors almost four decades ago in the early 1980s when several groups pioneered their use to express foreign antigens [66–72]. Since then, a number of veterinary vaccines based on different poxviruses have been commercially licensed, including the rabies vaccine. Rabies virus infection of humans is deadly, and most transmissions are through bites from infected animals. Recombinant vaccinia virus expressing rabies virus glycoprotein G has been successfully used to eliminate rabies from wildlife in some Western European countries [73–79]. In addition, ALVAC is a canarypox virus-based vector system, which has been used to develop several veterinary vaccines such as canine distemper virus, rabies virus, and equine influenza virus [80, 81]. Another veterinary vaccine, Trovac AI H5, is a fowlpox virus-based avian influenza virus vaccine expressing the avian influenza viral H5 antigen, which has been used in the United States and Central America [82]. Many human vaccines based on poxviruses, targeting various other infectious diseases, including HIV/AIDS, influenza, and Tuberculosis, are at different clinical or preclinical stages, comprehensively summarized by Sanchez-Sampedro et al. [83]. Many more poxvirus-based vaccines are under investigation. Recent efforts have focused on using Modified Vaccina virus Ankara to develop vaccines against SARS-CoV-2. These have shown promising effects in mice and macaques [84–87]. With further research, it is expected that poxvirus vector-based vaccines will be licensed for humans, which requires substantially increasing the investments and efforts to understand the fundamental aspects of poxvirus replication, as well as collaboration among poxvirologists, immunologists, clinicians, and veterinarians.

Oncolytic virotherapy is a targeted cancer therapy using viruses to infect and destroy cancer cells. While the idea to use viruses to treat cancers has been of interest to medical doctors for over a century, only in the past few decades has it become a highly promising and rapidly developing area. A growing number of patients benefit from this oncolytic therapy [88]. A herpesvirus-based oncolytic melanoma treatment, T-VEC, which met the US Food and Drug Administration (FDA) requirements in 2015, was the first treatment of this kind to gain approval [89]. There are a number of viruses with diverse characteristics in development to treat many kinds of cancers and poxviruses are among the most promising candidates for oncolytic actions. While several members of poxviruses from different genera (*Orthopoxvirus, Leporipoxvirus, and Yatapoxvirus)* have been the target of development, most research in the past decade has been focused on vaccinia virus (*Orthopoxvirus*) and myxoma virus (*Leporipoxvirus*) [90–95]. JX-594 was developed based on a modified vaccinia Copenhagen strain with deletion of viral thymidine kinase gene and insertion of granulocyte monocyte colony-stimulating factor (GM-CSF), which aimed to improve the ability of the virus to target and lyse cancer cells with specificity and stimulate anti-tumor immune responses. It was the first vaccinia virus-based oncolytic therapy that was tested in clinical trials starting with melanoma patients and was later engineered to combat other types of cancers [96–103]. More vaccinia virus strains have been developed and are in various stages of clinical or pre-clinical trials to treat hepatocellular carcinoma, pediatric solid tumors, lung cancer, etc. [104]. Interestingly, a non-replicating, inactivated modified vaccinia Ankara (MVA) has also been shown to have oncolytic potential [65], which would enhance the safety profiling of vaccinia virus-based oncolytic therapy. Another poxvirus with great potential in oncolytic research is myxoma virus [93, 105]. Myxoma viruses’ natural hosts are rabbits and so, advantageously, myxoma virus is non-replicating in normal human cells. However, studies have found that this virus can infect and kill non-rabbit cancer cells, including human cancer cells. This implies a robust safety profile for myxoma virus as it does not seem to do any harm, even to immunocompromised mice [93]. A recent review by Torres-Domínguez et al. comprehensively describes the progress in this field [104]. More development of poxviruses to fight different cancers in humans and animals is anticipated. Another critical aspect is to understand the functions of poxvirus genes in modulating the tumor microenvironment and anti-tumor immunity, which will allow genetic engineering of poxviruses to enhance their oncolytic specificity, potency, and safety profile.

## Conclusion remarks and perspectives

**Fig. 2:**
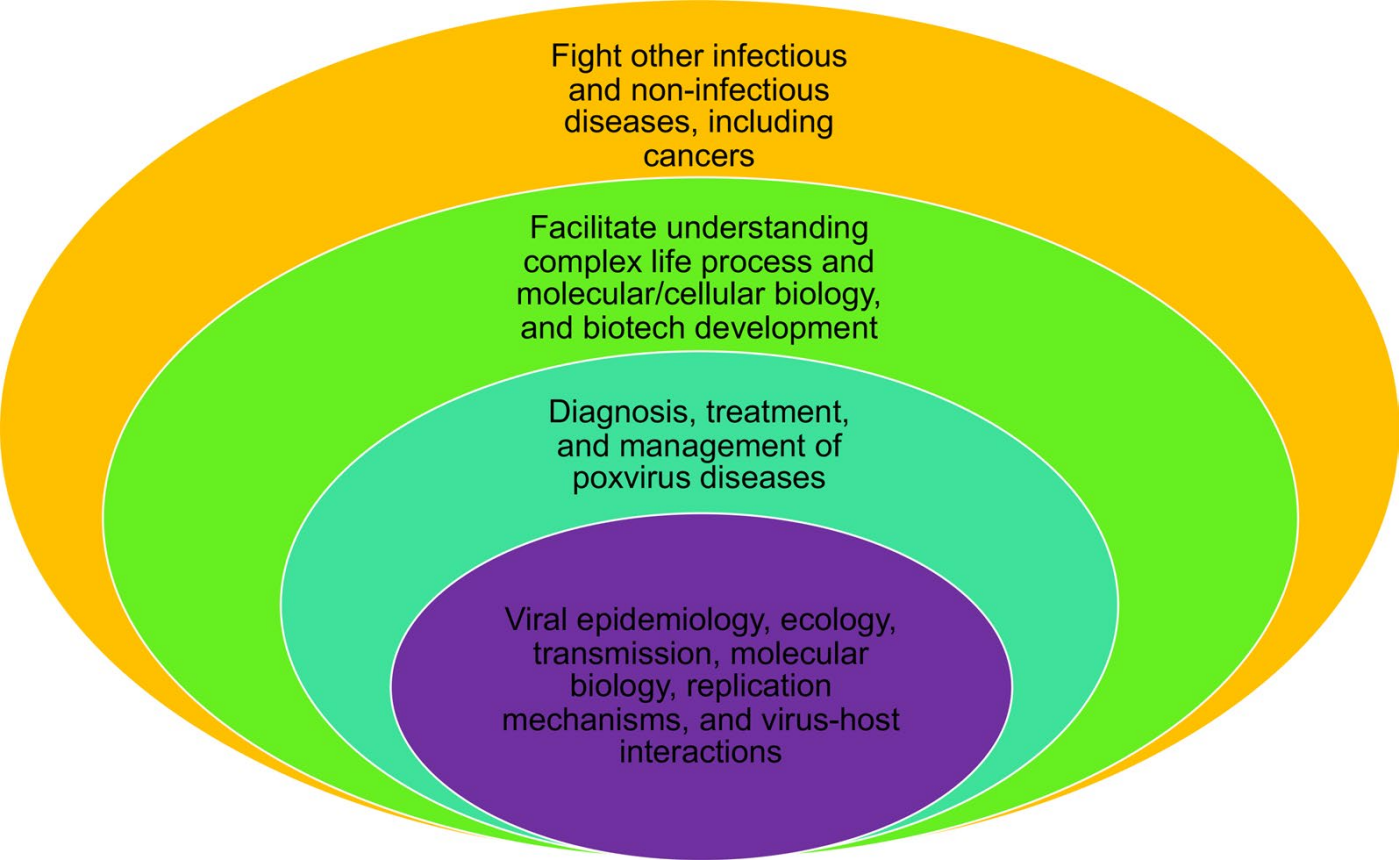
A perspective on poxvirus research

The poxvirus family includes many members that are pathogenic to humans and animals and pose significant risk to public health and economic activity. Understanding this large family of viruses, including their epidemiology, transmission, ecology, molecular biology, replication mechanisms, and strategies to take over the host cells, is the most critical task for developing the means to diagnose, treat, and manage these viruses; along with preventing future outbreaks. It is of note that almost all of these aspects of poxviruses are under-studied, and many questions remain unanswered. Poxviruses also possess tremendous scientific value for understanding life processes and cellular biology, thanks to their large genomes, complex virions, and life cycle. The utility of poxviruses in fighting other infectious diseases and cancers also has great potential. Improved understanding of this family of viruses will provide the foundation for genetically engineering them as better vaccine- and gene-delivery vectors and oncolytic agents. Figure 2 illustrates the aspects the author believes are critical in understating poxviruses and their utilities.

## Data Availability

Not applicable.
